# Supplementary material for: Assessing the dimensionality of scores derived from the Revised Formal Thought Disorder Self-Report Scale in schizotypy
Source: PLoS One. 2022 Dec 9;17(12):e0278841. doi: 10.1371/journal.pone.0278841 (PMC9733900; doi:10.1371/journal.pone.0278841)
Supplement: S1 File — (DOCX) [file pone.0278841.s001.docx]

**Supplemental Analyses and Considerations**

***The Impact of Excluding Items from the Exploratory Dimension-Reduction Analyses on Data Quality and the Resultant Factor Solutions***

The exploratory bifactor analysis identified several items that were potential candidates for removal. Specifically, two items (items 7 and 14) were not significant indicators of the general factor in Sample 1 and seven items (items 21, 22, 24, 25, 26, 28 and 29) were not significant indicators of any of the three group factors (see Table A). Removing the two items that did not load significantly on the general factor improved the sampling adequacy of the polychoric correlation matrix based on the Kaiser-Meyer-Olkin (KMO) index (KMO = 0.83, *CI*_95%_ [0.837, 0.849]) and Bartlett’s test of sphericity (χ^2^_[351]_ = 3605.5, *p* < 0.001). The parallel analysis still indicated the presence of three factors, though the factor solution still did not achieve simple structure when the exploratory factor analysis was completed (see Table C). Moreover, the removal of the seven items that were not significant indicators of any of the three group factors had much the same effect: sampling adequacy improved relative to the polychoric correlation matrix that encompassed all 29 items (KMO = 0.82 *CI*_95%_ [0.820, 0.856]; χ^2^_[231]_ = 3624.7, *p* < 0.001) and the parallel analysis indicated the presence of three factors, but the factor solution failed to achieve simple structure.

However, simple structure was essentially achieved with the removal of all nine items that failed to show strong factor loadings on both the general factor and one of the group factors in the bifactor model (see Table 2, main report), and this three-factor model was a good fit across most indices (see Table C). The solution explained 60.90% of the variance in the polychoric correlation matrix. Again, sampling adequacy was good (KMO = 0.85 *CI*_95%_ [0.873, 0.884]; χ^2^_[190]_ = 3632.3, *p* < 0.001) and the presence of three factors was supported by the parallel analysis. This 20-item version of the scale is referred to as the revised FTD-SS (FTD-SS-R).

There were no other obvious reasons to exclude items from the exploratory factor analysis in the current study. For instance, a review of the content of all 29 items did not reveal any obvious doublets. Indeed, collinearity was not immediately evident from the initial polychoric inter-item correlations in the current samples. In Sample 1, the largest correlations were between items 1 and 2 (*ρ* = 0.76, *CI*_95%_ [0.60, 0.85]), 9 and 10 (*ρ* = 0.74, *CI*_95%_ [0.52, 0.83]), 14 and 15 (*ρ* = 0.74, *CI*_95%_ [0.65, 0.85]), and 23 and 24 (*ρ* = 0.74, *CI*_95%_ [0.61, 0.84]). All remaining inter-item correlations were less than 0.70. Incidentally, this pattern of polychoric inter-item correlations was similar in Sample 2, particularly for items 1 and 2, and 9 and 10, although no effect size exceeded 0.70 in this sample. Nevertheless, using Ferrando et al.’s (2022) procedure based on residual correlations (implemented in FACTOR version 12.03.01), only items 9 and 10 were identified as possible doublets in Sample 1. Residual correlations derived from the Rasch analysis also indicated the removal of items 1 and 14 in addition to items 9 or 10.

As with the removal of the other items, the removal of items 1, 9 and 14 served to improve sampling adequacy (KMO = 0.82 *CI*_95%_ [0.836, 0.870]; χ^2^_[325]_ = 3609.3, *p* < 0.001). However, in contrast to the previous analyses, the removal of these items increased the evidence for unidimensionality. This was indicated by the parallel analysis, and all of the remaining items showed significant factor loadings on a single factor in the exploratory factor analysis. Yet, some items had very low communalities and only a minority of the variance in the polychoric correlation matrix was explained by the single factor (see Table C). Indeed, whilst most fit indices were strong, the root mean square of residuals (RMSR) was large. Furthermore, the removal of these items also improved the fit of the unidimensional model in the Rasch analysis (χ^2^ = 119.79, *p* = 0.138; PRI = 0.860; IRI = 0.915; PSI = 0.866; ISI = 0.892), and substantially improved the fit of the unidimensional model from the confirmatory factor analysis in Sample 2 (χ^2^ [299] = 946.320, *p* < 0.001; CFI = 0.919; TLI = 0.912; RMSEA = 0.060, *p* < 0.001; SRMR = 0.073; Average Item R^2^ = 0.386), although this fit remained inferior to the 20-item correlated three factors model.

***Assessing the Quality and Appropriateness of the Factor Estimates from the 20-Item Correlated Three Factors Model (Sample 1)***

The purpose of conducting factor analyses as part of a psychometric investigation is to illuminate the dimensions represented in the responses to the items on a measure. However, once a suitable factor solution has been found, this information must then be used to quantify an individual’s placement along those dimensions in a way which will ideally be generalizable to new samples. These measurements can be relatively unrefined, such as unweighted summed scores, or more refined, such as factor estimates based on linear regression (DiStefano et al., 2009; Ferrando & Lorenzo-Seva, 2018; Grice, 2001). Regardless of the specific method used, however, it has been argued that the scores derived from factor analyses should be evaluated in terms of their reliability and indeterminacy, correlational accuracy, and essential closeness to unidimensionality (Ferrando & Lorenzo-Seva, 2018; Grice, 2001).

Refined factor estimates derived from the three-factor solution of responses to the 20 items from the FTD-SS-R in Sample 1 were associated with large factor determinacy indices, indicating that the estimates were reliable. The factor estimates also exhibited inter-correlations (see Table D) that were very similar to the correlations between the factors themselves (see Table C), suggesting correlational accuracy. However, simple summed scores for each of the factors showed a very similar pattern of inter-correlations to those of the refined factor estimates (see Table E). Moreover, the correlation between the simple summed scores and the refined factor estimates were very close to 1.00 for all three factors (see Table F). Finally, two of the three closeness-to-unidimensionality indices still indicated overall essential unidimensionality (UniCo = 0.94, *CI*_95%_ [0.932, 0.967]; ECV = 0.80 *CI*_95%_ [0.771, 0.841]; MIREAL = 0.27 *CI*_95%_ [0.232, 0.300]). These assessments indicate that simple summed scores should suffice when using the FTD-SS-R, and also support the use of an overall FTD-SS-R total score.

| **Table A** |  | |  | |  |  |  |
| --- | --- | --- | --- | --- | --- | --- | --- |
| *Communalities, Estimated Factor Loadings and Inter-Factor Correlations for the Bifactor Model of Item Responses on the FTD-SS (Sample 1)* | | | | | | | |
| Item | Communality | | F1 | | F2 | F3 | GF |
| 1 | 0.73 | | **0.61** | | -0.22 | -0.08 | **0.61** |
| 2 | 0.61 | | **0.49** | | -0.23 | -0.04 | **0.61** |
| 3 | 0.67 | | **0.62** | | -0.04 | 0.07 | **0.52** |
| 4 | 0.50 | | **0.57** | | 0.05 | -0.08 | **0.42** |
| 5 | 0.46 | | **0.45** | | -0.06 | -0.07 | **0.52** |
| 6 | 0.57 | | **0.45** | | 0.17 | 0.05 | **0.54** |
| 7 | 0.38 | | 0.06 | | 0.17 | **0.48** | 0.24 |
| 8 | 0.63 | | 0.18 | | **0.51** | 0.05 | **0.52** |
| 9 | 0.71 | | -0.16 | | **0.70** | -0.15 | **0.49** |
| 10 | 0.73 | | -0.08 | | **0.72** | -0.13 | **0.49** |
| 11 | 0.51 | | 0.01 | | **0.40** | 0.09 | **0.57** |
| 12 | 0.47 | | 0.16 | | 0.10 | **0.33** | **0.53** |
| 13 | 0.48 | | 0.15 | | 0.00 | **0.37** | **0.53** |
| 14 | 0.62 | | -0.20 | | 0.12 | **0.78** | 0.15 |
| 15 | 0.69 | | -0.21 | | 0.21 | **0.74** | **0.32** |
| 16 | 0.68 | | 0.07 | | 0.12 | **0.57** | **0.53** |
| 17 | 0.37 | | -0.21 | | -0.14 | **0.51** | **0.35** |
| 18 | 0.50 | | 0.01 | | 0.00 | **0.46** | **0.54** |
| 19 | 0.51 | | -0.11 | | 0.11 | **0.56** | **0.42** |
| 20 | 0.45 | | -0.14 | | **0.33** | 0.16 | **0.55** |
| 21 | 0.53 | | 0.27 | | -0.03 | 0.06 | **0.67** |
| 22 | 0.53 | | 0.13 | | 0.25 | 0.27 | **0.57** |
| 23 | 0.65 | | -0.17 | | -0.26 | **0.32** | **0.71** |
| 24 | 0.66 | | -0.11 | | -0.17 | 0.26 | **0.76** |
| 25 | 0.41 | | -0.02 | | 0.05 | 0.18 | **0.61** |
| 26 | 0.50 | | 0.11 | | 0.24 | 0.01 | **0.65** |
| 27 | 0.55 | | 0.06 | | -0.10 | **0.32** | **0.66** |
| 28 | 0.52 | | -0.10 | | -0.09 | 0.24 | **0.68** |
| 29 | 0.48 | | -0.01 | | 0.25 | 0.18 | **0.61** |
|  | Mean | | 0.49 | | 0.45 | 0.43 | 0.53 |
| Inter-Factor Correlations | |  | |  |  |  |  |
|  | F1 | | - | |  |  |  |
|  | F2 | | 0.20 | | - |  |  |
|  | F3 | | 0.30 | | 0.22 | - |  |
|  | GF | | 0.00 | | 0.00 | 0.00 | - |
| *Note.* Factor loadings > 0.30 are presented in bold. Underlined values represent non-significant factor loadings included in the calculated means. FTD-SS factor labels: F1 - Difficulty with Maintaining the Topic of Conversation; F2 - Difficulty with Initiating and Sustaining Speech; F3 - Odd Speech; GF – General (DCTS) Factor. | | | | | | | |

| **Table B** | |  | |  | |  | |  | |  | |  |
| --- | --- | --- | --- | --- | --- | --- | --- | --- | --- | --- | --- | --- |
| *Closeness-to-Unidimensionality Indices for Each Item and Overall for the Exploratory Bifactor Model of FTD-SS Responses (Sample 1)* | | | | | | | | | | | |  |
| Item | UniCo | | (*CI*_95%_) | | ECV | | (*CI*_95%_) | | IREAL | | (*CI*_95%_) | |
| 1 | 0.76 | | (0.420, 0.958) | | 0.54 | | (0.317, 0.770) | | 0.58 | | (0.443, 0.752) | |
| 2 | 0.83 | | (0.452, 0.990) | | 0.60 | | (0.336, 0.877) | | 0.49 | | (0.275, 0.657) | |
| 3 | 0.94 | | (0.694, 0.995) | | 0.73 | | (0.491, 0.906) | | 0.40 | | (0.250, 0.565) | |
| 4 | 0.80 | | (0.374, 0.931) | | 0.57 | | (0.287, 0.718) | | 0.47 | | (0.394, 0.593) | |
| 5 | 0.86 | | (0.311, 0.969) | | 0.63 | | (0.246, 0.797) | | 0.43 | | (0.331, 0.616) | |
| 6 | 0.98 | | (0.840, 1.000) | | 0.85 | | (0.608, 0.977) | | 0.29 | | (0.134, 0.479) | |
| 7 | 0.94 | | (0.282, 0.999) | | 0.73 | | (0.227, 0.953) | | 0.31 | | (0.172, 0.537) | |
| 8 | 1.00 | | (1.000, 1.000) | | 1.00 | | (0.989, 1.000) | | 0.02 | | (0.000, 0.069) | |
| 9 | 1.00 | | (0.858, 1.000) | | 0.96 | | (0.626, 1.000) | | 0.10 | | (0.000, 0.389) | |
| 10 | 1.00 | | (0.974, 1.000) | | 0.98 | | (0.810, 1.000) | | 0.07 | | (0.000, 0.263) | |
| 11 | 1.00 | | (0.996, 1.000) | | 0.99 | | (0.916, 1.000) | | 0.05 | | (0.000, 0.200) | |
| 12 | 1.00 | | (0.999, 1.000) | | 1.00 | | (0.962, 1.000) | | 0.04 | | (0.000, 0.137) | |
| 13 | 1.00 | | (0.999, 1.000) | | 1.00 | | (0.968, 1.000) | | 0.04 | | (0.000, 0.120) | |
| 14 | 0.48 | | (0.085, 0.857) | | 0.35 | | (0.078, 0.624) | | 0.61 | | (0.509, 0.790) | |
| 15 | 0.74 | | (0.244, 0.923) | | 0.53 | | (0.201, 0.706) | | 0.57 | | (0.486, 0.821) | |
| 16 | 1.00 | | (0.941, 1.000) | | 0.91 | | (0.735, 0.982) | | 0.25 | | (0.130, 0.416) | |
| 17 | 0.85 | | (0.215, 0.998) | | 0.62 | | (0.180, 0.940) | | 0.34 | | (0.141, 0.618) | |
| 18 | 1.00 | | (0.945, 1.000) | | 0.94 | | (0.743, 0.999) | | 0.17 | | (0.025, 0.379) | |
| 19 | 0.95 | | (0.624, 0.998) | | 0.75 | | (0.444, 0.942) | | 0.36 | | (0.163, 0.585) | |
| 20 | 1.00 | | (0.919, 1.000) | | 0.92 | | (0.699, 1.000) | | 0.18 | | (0.011, 0.368) | |
| 21 | 0.99 | | (0.886, 0.998) | | 0.86 | | (0.657, 0.937) | | 0.28 | | (0.207, 0.435) | |
| 22 | 1.00 | | (0.996, 1.000) | | 0.99 | | (0.919, 1.000) | | 0.06 | | (0.002, 0.209) | |
| 23 | 1.00 | | (0.993, 1.000) | | 0.99 | | (0.891, 1.000) | | 0.06 | | (0.000, 0.223) | |
| 24 | 1.00 | | (1.000, 1.000) | | 1.00 | | (1.000, 1.000) | | 0.01 | | (0.000, 0.011) | |
| 25 | 1.00 | | (1.000, 1.000) | | 1.00 | | (1.000, 1.000) | | 0.00 | | (0.000, 0.001) | |
| 26 | 1.00 | | (0.937, 1.000) | | 0.96 | | (0.728, 1.000) | | 0.14 | | (0.020, 0.370) | |
| 27 | 1.00 | | (1.000, 1.000) | | 1.00 | | (1.000, 1.000) | | 0.00 | | (0.000, 0.000) | |
| 28 | 1.00 | | (1.000, 1.000) | | 1.00 | | (0.971, 1.000) | | 0.03 | | (0.000, 0.109) | |
| 29 | 1.00 | | (0.994, 1.000) | | 0.99 | | (0.902, 1.000) | | 0.07 | | (0.003, 0.228) | |
| Overall | 0.93 | | (0.889, 0.954) | | 0.82 | | (0.768, 0.847) | | 0.22 | | (0.201, 0.262) | |
| *Note.* UniCo values larger than 0.95, ECV values larger than 0.85 and IREAL values less than 0.30 indicate essential unidimensionality (Ferrando & Lorenzo-Seva, 2018). Abbreviations: ECV - Explained Common Variance Index; IREAL – Item Residual Absolute Loadings; UniCo – Unidimensional Congruence | | | | | | | | | | | |  |

| **Table C** |  |  |  |  |
| --- | --- | --- | --- | --- |
| *Systematic Order of the Exploratory Dimension Reduction Analyses Conducted, Including Repeated Exploratory Factor Analyses Conducted after the Successive Removal of FTD-SS Items (Sample 1)* | | | | |
| Analysis  (Estimation and Rotation) | Items Removed | Number of Factors | Robust Fit Indices | Comments |
| MRFA, Promin | None | 3 | n/a | Inadmissible factor solution (six Heywood cases with item communalities of 1.00) |
| ULS, Promin | None | 3 | χ^2^_(322)_ = 489.01, *p* < 0.001  BIC = 1159.58, *CI*_95%_ (1072.324, 1180.809)  CFI = 0.99, *CI*_95%_ 0.989, 0.994)  TLI = 0.99, *CI*_95%_ (0.989, 0.994)  GFI = 0.98, *CI*_95%_ (0.980, 0.986)  RMSEA = 0.04, *CI*_95%_ (0.028, 0.043)  RMSR = 0.06, *CI*_95%_ (0.053, 0.061) | Appropriate item communalities (no Heywood cases); simple structure not achieved (six items exhibited cross-loadings); strong inter-factor correlations (*r* ≥ 0.61) |
| (EBFA) ULS, Promin | None | 4 | n/a | Mixed evidence for and against essential unidimensionality. |
| ULS, Promin | 7, 14 | 3 | χ^2^_(273)_ = 409.55, *p* < 0.001  BIC = 1033.87, *CI*_95%_ (952.250, 1058.380)  CFI = 0.99, *CI*_95%_ (0.992, 0.997)  TLI = 0.99, *CI*_95%_ (0.989, 0.996)  GFI = 0.98, *CI*_95%_ (0.980, 0.986)  RMSEA = 0.04, *CI*_95%_ (0.025, 0.043)  RMSR = 0.06, *CI*_95%_ (0.049, 0.057) | Appropriate item communalities (no Heywood cases); simple structure not achieved (four items exhibited cross-loadings) |
| ULS, Promin | 21, 22, 24, 25, 26, 28, 29 | 3 | χ^2^_(168)_ = 273.11, *p* < 0.001  BIC = 781.82, *CI*_95%_ (701.934, 812.878)  CFI = 0.99, *CI*_95%_ (0.987, 0.997)  TLI = 0.99, *CI*_95%_ (0.982, 0.996)  GFI = 0.99, *CI*_95%_ (0.983, 9.990)  RMSEA = 0.04, *CI*_95%_ (0.022, 0.050)  RMSR = 0.06, *CI*_95%_ (0.045, 0.057) | Appropriate item communalities (no Heywood cases); simple structure not achieved (three items exhibited cross-loadings) |
| **ULS, Promin** | **7, 14, 21, 22, 24, 25, 26, 28, 29** | **3** | **χ^2^_(133)_ = 222.17, *p* < 0.001**  **BIC = 684.63, *CI*_95%_ (598.545, 712.684)**  **CFI = 0.99, *CI*_95%_ (0.987, 1.000)**  **TLI = 0.99, *CI*_95%_ (0.982, 0.999)**  **GFI = 0.99, *CI*_95%_ (0.986, 0.992)**  **RMSEA = 0.05, *CI*_95%_ (0.009, 0.052)**  **RMSR = 0.05, *CI*_95%_ (0.039, 0.054)** | **Three factors explained 60.90% of the variance in the polychoric correlation matrix; simple structure achieved (only one item showed cross-loadings; these loadings were in opposite directions, with one being of marginal significance)** |
|  |  |  |  |  |

| ULS | 1, 9, 14 | 1 | χ^2^_(299)_ = 737.37, *p* < 0.001  BIC = 1037.97, *CI*_95%_ (820.858, 1122.854)  CFI = 0.97, *CI*_95%_ (0.969, 0.986)  TLI = 0.97, *CI*_95%_ (0.966, 0.985)  GFI = 0.96, *CI*_95%_ (0.953, 0.970)  RMSEA = 0.07, *CI*_95%_ (0.048, 0.074)  RMSR = 0.12, *CI*_95%_ (0.095, 0.132) | Communalities < 0.3 for five items, and particularly low for item 17; one factor explained 41.75% of the variance in the polychoric correlation matrix |
| --- | --- | --- | --- | --- |

| *Note.* Bold-face highlights the best factor solution. Abbreviations: BIC – Schwarz’s Bayesian Information Criterion; CFI – Comparative Fit Index; EBFA – Exploratory Bifactor Analysis; MRFA – Minimum Rank Factor Analysis; TLI – Tucker-Lewis Index; RMSEA – Root Mean Square Error of Approximation; RMSR –Root Mean Square of Residuals; ULS – Unweighted Least Squares |
| --- |

| **Table D** |  | |  | | |  | |  | | | |
| --- | --- | --- | --- | --- | --- | --- | --- | --- | --- | --- | --- |
| *Spearman’s Inter-Correlations and Factor Determinacy Indices for Regression-Based FTD-SS-R Factor Estimates (Sample 1)* | | | | | | | | | | | |
|  | | F1 | | | F2 | | | | F3 | | |
| F1 | | - | | |  | | | |  | | |
| F2 | | 0.44*** | | | - | | | |  | | |
| F3 | | 0.59*** | | | 0.52*** | | | | - | | |
| Factor Determinacy Index  (*CI*_95%_) | | 0.96  (0.942, 0.969) | | | 0.95  (0.923, 0.975) | | | | 0.95  (0.937, 0.965) | | |
| *Note.* *** *p* < 0.001 (uncorrected for multiple tests). Factor estimates are Fully-Informative Prior Oblique Expected A Posteriori linear estimates (Ferrando & Lorenzo-Seva, 2016, 2018). Factor labels: F1 - Difficulty with Maintaining the Topic of Conversation; F2 - Difficulty with Initiating and Sustaining Speech; F3 - Odd Speech. | | | | | | | | | | | |
| **Table E** |  | |  | | |  | |  | | | |
| *Spearman’s Inter-Correlations for FTD-SS-R Simple Summed Scores Obtained (Sample 1)* | | | | | | | | | | | |
|  | | F1 | | F2 | | | F3 | | | Total (adj.) | |
| F1 | | - | |  | | |  | | |  | |
| F2 | | 0.41*** | | - | | |  | | |  | |
| F3 | | 0.46*** | | 0.46*** | | | - | | |  | |
| Total (adjusted) | | 0.51*** | | 0.51*** | | | 0.54*** | | | - | |
| *Note.* *** *p* < 0.001 (uncorrected for multiple tests). Factor labels: F1 - Difficulty with Maintaining the Topic of Conversation; F2 - Difficulty with Initiating and Sustaining Speech; F3 - Odd Speech. Simple sum scores were an unweighted sum of item responses for items that loaded significantly onto the factor. Adjusted total scores were the sum of item responses for all items not included in the factor that it is being correlated with. | | | | | | | | | | | |
| **Table F** |  | |  | | |  | |  | | |  |
| *Spearman’s Correlations between Regression-Based FTD-SS-R Factor Estimates and Simple Summed Scores (Sample 1)* | | | | | | | | | | | |
|  | | Regression-Based FTD-SS Factor Estimates | | | | | | | | | |
| Simple Summed Scores | | F1 | | | F2 | | | | F3 | | |
| F1 | | 0.97*** | | | 0.39*** | | | | 0.53*** | | |
| F2 | | 0.47*** | | | 0.95*** | | | | 0.53*** | | |
| F3 | | 0.53*** | | | 0.47*** | | | | 0.97*** | | |
| Total | | 0.82*** | | | 0.74*** | | | | 0.87*** | | |
| *Note.* *** *p* < 0.001 (uncorrected for multiple tests). Factor estimates are Fully-Informative Prior Oblique Expected A Posteriori linear estimates (Ferrando & Lorenzo-Seva, 2016, 2018). Factor labels: F1 - Difficulty with Maintaining the Topic of Conversation; F2 - Difficulty with Initiating and Sustaining Speech; F3 - Odd Speech. | | | | | | | | | | | |

| **Table G** | | | |
| --- | --- | --- | --- |
| *Standardized Factor Loadings and Covariances, and Robust Standard Errors for the Three-Factor Model of Responses to the 20-Item FTD-SS-R Investigated Using Confirmatory Factor Analysis (Sample 2)* | | | |
|  |  | Standardized Loadings | Robust Standard Errors |
| F1 | Item 1 | 0.73*** | 0.03 |
|  | Item 2 | 0.68*** | 0.03 |
|  | Item 3 | 0.71*** | 0.03 |
|  | Item 4 | 0.74*** | 0.03 |
|  | Item 5 | 0.68*** | 0.03 |
|  | Item 6 | 0.69*** | 0.03 |
| F2 | Item 8 | 0.79*** | 0.03 |
|  | Item 9 | 0.67*** | 0.04 |
|  | Item 10 | 0.71*** | 0.04 |
|  | Item 11 | 0.79*** | 0.03 |
|  | Item 20 | 0.67*** | 0.04 |
| F3 | Item 12 | 0.74*** | 0.03 |
|  | Item 13 | 0.63*** | 0.04 |
|  | Item 15 | 0.69*** | 0.04 |
|  | Item 16 | 0.78*** | 0.03 |
|  | Item 17 | 0.45*** | 0.05 |
|  | Item 18 | 0.74*** | 0.03 |
|  | Item 19 | 0.68*** | 0.04 |
|  | Item 23 | 0.68*** | 0.03 |
|  | Item 27 | 0.57*** | 0.04 |
| F1 | F2 | 0.71*** | 0.03 |
|  | F3 | 0.76*** | 0.03 |
| F2 | F3 | 0.57*** | 0.04 |
| *Note.* *** *p* < 0.001. Factor labels: F1 - Difficulty with Maintaining the Topic of Conversation; F2 - Difficulty with Initiating and Sustaining Speech; F3 - Odd Speech. | | | |

| **Table H** | | | |
| --- | --- | --- | --- |
| *Pearson’s Product-Moment Correlations between FTD-SS-R Summed Scores (Transformed) Derived from the 20-Item Three Correlated Factors Model, and Scores Derived from Other Measures of Schizotypy and Personality in the Combined Participant Sample (N = 926)* | | | |
|  | FTD-SS-R (20 Items) | | |
|  | F1 | F2 | F3 |
|  |  | | |
| O-LIFE Cognitive Disorganization | 0.55*** | 0.56*** | 0.47*** |
| O-LIFE Unusual Experiences | 0.41*** | 0.33*** | 0.52*** |
| O-LIFE Introvertive Anhedonia | 0.26*** | 0.47*** | 0.24*** |
| O-LIFE Impulsive Non-Conformity | 0.31*** | 0.16*** | 0.40*** |
| SPQ Ideas of Reference | 0.38*** | 0.29*** | 0.45*** |
| SPQ Excessive Social Anxiety | 0.35*** | 0.60*** | 0.26*** |
| SPQ Odd Beliefs or Magical Thinking | 0.17*** | 0.09*** | 0.26*** |
| SPQ Unusual Perceptual Experiences | 0.34*** | 0.26*** | 0.45*** |
| SPQ Odd or Eccentric Behaviour | 0.29*** | 0.30*** | 0.49*** |
| SPQ No Close Friends | 0.29*** | 0.59*** | 0.27*** |
| SPQ Odd Speech | 0.57*** | 0.41*** | 0.63*** |
| SPQ Constricted Affect | 0.29*** | 0.57*** | 0.33*** |
| SPQ Suspiciousness | 0.38*** | 0.36*** | 0.37*** |
| IPIP Neuroticism | 0.43*** | 0.45*** | 0.33*** |
| IPIP Extraversion | -0.21*** | -0.58*** | -0.15*** |
| *Note.* *** *p* < .001. Probability values for Pearson’s product-moment correlations were adjusted for multiple tests using the false discovery rate (Benjamin & Hochberg, 1995). Factor labels: F1 - Difficulty with Maintaining the Topic of Conversation; F2 - Difficulty with Initiating and Sustaining Speech; F3 - Odd Speech. Abbreviations: FTD-SS-R – Formal Thought Disorder – Self Scale – Revised; IPIP – International Personality Item Pool; O-LIFE – Oxford-Liverpool Inventory of Feelings and Experiences; SPQ – Schizotypal Personality Questionnaire | | | |

| **Table I** | | |  |  | |  |
| --- | --- | --- | --- | --- | --- | --- |
| *Comparisons of Total Scores (Transformed) from the Original 29-Item FTD-SS across Demographic Variables in the Combined Participant Sample* | | | | | | |
|  | *n* | Mean (*SD*) | | | Significant Pairwise Comparisons^a^ | |
| Gender (*F*_[1, 928]_ = 4.78, *p* = 0.03, ω^2^ = 0.00, *CI*_95%_ [0.000, 0.016]) | | | | | | |
| Male (M) | 297 | 45.64 (11.91) | | | M > F; *g* = 0.15, *CI*_95%_ (0.016, 0.292) | |
| Female (F) | 633 | 43.70 (10.35) | | |  | |
| Country of Birth (*F*_[1, 930]_ = 2.17, *p* = 0.14, ω^2^ = 0.00, *CI*_95%_ [0.000, 0.010]) | | | | | | |
| Australia | 710 | 44.58 (10.88) | | |  | |
| Other | 222 | 43.54 (10.97) | | |  | |
| Highest Level of Completed Education (*F*_[3, 922]_ = 4.30, *p* = 0.01, ω^2^ = 0.01, *CI*_95%_ [0.001, 0.025]) | | | | | | |
| Secondary school (S) | 297 | 45.53 (11.22) | | | S > P; *g* = 0.43, *CI*_95%_ (0.180, 0.674) | |
| Technical or vocational training (T) | 320 | 44.48 (11.10) | | | T > P; *g* = 0.33, *CI*_95%_ (0.083, 0.571) | |
| Undergraduate university degree | 227 | 43.46 (10.05) | | |  | |
| Postgraduate university degree (P) | 82 | 41.60 (10.92) | | |  | |
| Student Status (*F*_[2, 931]_ = 3.47, *p* = 0.03, ω^2^ = 0.01, *CI*_95%_ [0.000, 0.017]) | | | | | | |
| Full-time (Ft) | 420 | 45.03 (11.66) | | | Ft > Pt; *g* = 0.16, *CI*_95%_ (0.018, 0.301) | |
| Part-time (Pt) | 356 | 42.92 (9.39) | | | Pt < N; *g* = -0.22, *CI*_95%_ (-0.406, -0.030) | |
| Not a student (N) | 158 | 45.58 (11.66) | | |  | |
| Employment Status (*F*_[4, 929]_ = 3.56, *p* = 0.01, ω^2^ = 0.01, *CI*_95%_ [0.000, 0.024]) | | | | | | |
| Unemployed (Un) | 147 | 46.20 (10.71) | | | Un > Ft; *g* = 0.37, *CI*_95%_ (0.176, 0.572) | |
| Casual or part-time | 322 | 44.60 (11.51) | | |  | |
| Full-time (Ft) | 312 | 42.81 (10.04) | | |  | |
| Self-employed | 50 | 43.36 (9.14) | | |  | |
| Other or mixed | 103 | 45.80 (11.93) | | |  | |
| Past Mental Health Diagnosis (*F*_[1, 200.85]_ = 5.47, *p* = 0.02, ω^2^ = 0.02, *CI*_95%_ [0.000, 0.076]) | | | | | | |
| Yes (Y) | 138 | 45.79 (10.89) | | | Y > N; *g* = 0.20, *CI*_95%_ (0.019, 0.381) | |
| No (N) | 796 | 44.06 (10.88) | | |  | |
| Immediate Family Member with a Schizophrenia-Related Disorder (*F*_[2, 931]_ = 3.72, *p* = 0.02, ω^2^ = 0.01, *CI*_95%_ [0.000, 0.018]) | | | | | | |
| Yes | 56 | 46.38 (12.11) | | |  | |
| No (N) | 821 | 43.94 (10.60) | | | N < U; *g* = -0.32, *CI*_95%_ (-0.586, -0.048) | |
| Unsure (U) | 57 | 47.75 (13.00) | | |  | |
| *Note*. Only four participants identified their gender as non-binary and only eight participants completed primary school as their highest level of education. Thus, these responses were omitted from the current analyses. Transformed FTD-SS total scores (negative reciprocal transformation, total score calculated across all 29 items) were the dependent variable in all analyses.  ^a^ Probability values for post-hoc tests (i.e. Tukey’s HSD or Games-Howell tests) were adjusted for familywise error using the Bonferroni method, with an alpha criterion of *p*_adj_ *≤* .05 (underlined: 0.05 < *p*_adj_ < .10). Hedge’s *g* effect sizes provided for significant (or near-significant) pairwise comparisons. Post-hoc tests only performed for variables exhibiting significant main effects. | | | | | | |

**References**

Benjamin, Y., & Hochberg, Y. (1995). Controlling the False Discovery Rate: A Practical and Powerful Approach to Multiple Testing. *Journal of the Royal Statistical Society. Series B (Methodological)*, *57*(1), 289-300. <https://www.jstor.org/stable/2346101>

DiStefano, C., Zhu, M., & Mindrila, D. (2009). Understanding and using factor scores: Considerations for the applied researcher. *Practical Assessment, Research & Evaluation*, *14*(20). <https://doi.org/10.7275/da8t-4g52>

Ferrando, P. J., Hernandez-Dorado, A., & Lorenzo-Seva, U. (2022). Detecting Correlated Residuals in Exploratory Factor Analysis: New Proposals and a Comparison of Procedures. *Structural Equation Modeling: A Multidisciplinary Journal*, *29*(4), 630-638. <https://doi.org/10.1080/10705511.2021.2004543>

Ferrando, P. J., & Lorenzo-Seva, U. (2016). A note on improving EAP trait estimation in oblique factor-analytic and item response theory models. *Psicologica*, *37*, 235-247.

Ferrando, P. J., & Lorenzo-Seva, U. (2018). Assessing the Quality and Appropriateness of Factor Solutions and Factor Score Estimates in Exploratory Item Factor Analysis. *Educational and Psychological Measurement*, *78*(5), 762-780. <https://doi.org/10.1177/0013164417719308>

Grice, J. W. (2001). Computing and evaluating factor scores. *Psychological Methods*, *6*(4), 430-450. <https://doi.org/10.1037//1082-989x.6.4.430>
