# Supplementary material for: Assessing the dimensionality of scores derived from the Revised Formal Thought Disorder Self-Report Scale in schizotypy
Source: PLoS One. 2022 Dec 9;17(12):e0278841. doi: 10.1371/journal.pone.0278841 (PMC9733900; doi:10.1371/journal.pone.0278841)
Supplement: Attachment — Submitted filename: Response to Reviewers.docx [file pone.0278841.s003.docx]

Prof. Marco Innamorati

Academic Editor

PLOS ONE

Resubmission of manuscript ‘*Assessing the dimensionality of scores derived from the Formal Thought Disorder Self-Report Scale in schizotypy*’

16^th^ December, 2021

Dear Professor Marco,

We would like to thank you for the opportunity to revise our manuscript, and to thank the reviewers for their thoughtful and constructive feedback. It is our opinion that the research has been improved as a result, and we hope it will eventuate in a useful contribution to the literature.

Please find our responses to each of the reviewers’ comments below. For clarity, we have presented the comments in bold and in italics, and included a description of how and where (i.e. line numbers) the text was modified. As requested, changes made in the manuscript are marked using tracked changes. The amendments were discussed and agreed upon by all four authors.

We eagerly await your reply regarding our revisions and are ready to respond to any further comments and questions that you may have.

Sincerely,

Philip Sumner, PhD

Swinburne University of Technology

Note. Line and page numbers are based on the manuscript displayed with tracked changes using “All Markup”.

**Reviewer #1*:***

***The aim of the manuscript was to assess the dimensionality of the "Formal Thought Disorder Self- Report Scale. Although the topic of the manuscript seems to be of scientific interest, here are some comments which could improve the quality of the manuscript.***

Thank you for your feedback!

***- I kindly ask the authors to add to the description of the FTD-SS what lower and higher score reflect (e.g., greater difficulties with communication?).***

We have added this into the description of the FTD-SS on page 8 (lines 158-160), indicating that higher scores reflect more frequent difficulties with communication.

***- Something with the samples is not fully convincing. The authors state that “The FTD-SS was presented as part of a larger online survey.”***

***So, it seems legit to ask whether these samples were in reality one big sample which was splitted into three? If this is the case, I kindly ask the authors to repeat the analyses on the whole sample and compare the different models fit using the nested model approach, because I cannot see the point of splitting the sample. If this is not the case, then I would like to see whether there are any statistical significant differences in socio-demographics among these samples.***

As mentioned in the introduction (pages 6-7, lines 120-127), only one study had previously investigated the dimensionality of non-clinical disorganised or constrained thought and speech (DCTS), and this study used a Principal Components Analysis (Barrera et al., 2015). Moreover, the dimensionality of clinical DCTS is not yet agreed upon. Thus, because there was no theoretical basis to guide a CFA, we initially set out to investigate the dimensionality of the FTD-SS using exploratory (i.e. data-driven) analyses based on polychoric correlations (as appropriate for ordinal data).

Since the bifactor model from the EFA of Study 1 diverged somewhat from Barrera et al.’s (2015) correlated three-factors model, we decided to continue data collection so that we could use CFA to assess the replicability of the model in a new sample, and so that we could determine the improvement in fit over competing models. As such, the aim of Study 2 was motivated directly from the findings of Study 1, and it would not be appropriate to combine the samples/analysis between Study 1 and Study 2.

This approach has been advocated as good model development practice (Byrne, 2010). In particular, EFA should be used when the links between observed and latent variables are uncertain, followed by CFA to validate the measurement model suggested by the EFA (pg. 5, Byrne, 2010). Using separate samples for the EFA and CFA ensures that there is a robust validation process.

On the other hand, the recruitment of the third sample was done in an effort to improve the generalisability of the current findings by being less reliant upon student participants. Although we managed to recruit more non-students and more males through Prolific, the sampled FTD-SS scores (and levels of schizotypy more generally) were highly similar. Therefore, in-light of your feedback, we have re-conducted the CFA combining the second and third samples (now referred to as “Sample 2”). The limits to generalisability are still mentioned in the discussion.

***- Line 193: Add the values of kurtosis and skewness***

These values for each item have been added to Table 2, and this addition has been referred to in-text (page 15, lines 198-199).

***- Line 222: I did not understand whether these 9 items (the 7 items which did not load significantly on any of the three factors and the 2 items which did not load on the common factor), where still included in the scale and further analyses? If yes, please explain which item were kept (just the 2 items, or the 7 items, or all the 9 items), and why this decision was made.***

All 9 items were retained in the subsequent CFA in Study 2. There were several reasons for this. Firstly, as mentioned in the materials section of Study 1 (page 8, lines 152-156), all 29 items from the FTD-SS were specifically designed based on distinct clinical symptoms of thought disorder, and so are important conceptually. Moreover, the items were retained from an initial pool of 52 items based on evidence of their reliability and level of endorsement in a clinical sample of people with schizophrenia (Barrera et al., 2008). These items, including the 9 in question here, were found to load significantly in the factor solution reported for this clinical sample, as well as in the correlated three-factors model reported by Barrera et al. (2015) in their non-clinical sample. It is also conceptually important to replicate the factor loadings for these 9 items because non-significant loadings might mean that these indicators are less important in non-clinical samples than in people with schizophrenia. Hence, we were reluctant to remove items without first confirming these loadings in the second sample.

Moreover, one of the aims of the CFA in Study 2 was to compare the bifactor model with other models, including Barrera et al.’s (2015) model, and this requires the retention of all 29 items, with weak/non-significant loadings attenuating support for a model. The 2 items that did not load significantly on the general factor might be considered more problematic for the bifactor model because of the importance/dominance of the general factor (based on the closeness-to-unidimensionality indices from the EFA provided in the supporting information section). Nonetheless, in the CFA, we found that all 29 items (including items 7 and 14) loaded significantly on the bifactor model, and we confirmed that dominance of the general factor. Thus, these items were included in the total scores for the regression analyses of Study 3.

Ultimately, there might be an argument for the removal of items from the FTD-SS as, in addition to the factor loadings, the large internal consistency coefficients in the current samples could indicate redundancy. However, the removal of particular items here would be premature because they depend on the theoretical interpretation of the bifactor model. For example, if the group factors represent nuisance variables unrelated to DCTS, then the 7 items that do not load significantly on any of the group factors might be considered better indicators of DCTS than the remaining items. However, if the group factors represent theoretically meaningful dimensions of DCTS, then these items are also important and might be considered better indicators of their group factors. These possibilities illustrate the importance of determining the convergent validity of the factors, which we began to do in Study 3. Nevertheless, further research is required, as we have indicated in the general discussion (page 40-41).

***- Moreover, it is not clear whether the three factors identified in your model, reflect the structure of the factors reported by Barrera? In other words, the items loading on your factors, also load together in the model identified by Barrera? If not, please what could be a possible explanation and why did you change the name of the factors? Finally, please adapt the discussion on the basis of these results.***

Twenty-two items showed significant group factor loadings within the bifactor model. With one exception (item 4), these items showed significant loadings on the same factors as those reported by Barrera et al. (2015). As alluded to in the discussion (page 19, line 263-265), the main difference was that the introduction of the general factor resulted in 7 items that no longer loaded significantly on any group factor. Notably, these 7 items tended to be amongst those with the lowest factor loadings in Barrera et al.’s (2015) correlated three-factors model.

Despite these similarities, we elected to change the factor labels from those of Barrera et al. (2015) in favour of more descriptive terms, in-line with our previous work (Sumner et al., 2020). Barrera et al. (2015) themselves were inconsistent in their labelling of factor 2, which they referred to both as “Alogia” and “Conversational Ability”. Moreover, their use of “Working Memory Deficit” implies that the factor results from issues with working memory function, which has not yet been established. We have now explained this re-labelling in the results section of Study 1 (page 16, lines 233-236).

***- Please move “Study 2” before the aim of the study because the readers cannot tell where the discussion of the previous study end and where the aim of the new study begin.***

We have moved the title, as suggested.

**- Line 322: I do not fully understand the rationale behind testing a second-order model on the bifactor model. Could the authors explain this decision?**

Indeed, the two models appear similar, both representing three group factors and a general factor. However, the two models differ in the specified relationships between the group and general factors, and so yield different conceptual representations of multidimensionality (see Reise et al., 2010). In the bifactor model, direct relationships are modelled between the observed item variance and the general factor, and the group factors explain additional item variance that is not accounted for by the general factor. One intuitive interpretation of the group factors is that they represent nuisance variables that interfere with the measurement of the general factor (Reise et al., 2010). By contrast, in the second-order hierarchical model, the general factor is not directly related to the item variances. Instead, the general factor accounts for the variance that is common to the group factors.

We attempted to explain this distinction in the discussion for Study 1 using intelligence models as an example. The explanation has been re-worded slightly in an effort to clarify the rationale (page 21, lines 301-305, 310-312). The differences between the models are also depicted in Figures 3 and 4.

***- Line 344: The authors state “Notably, however, none of the participants in Sample 2 endorsed the fourth response category for item 15. The distribution for the item was similar in Sample 3, with only three participants endorsing the fourth response category. Thus, for comparability, the third and fourth response categories for this item were collapsed together in Sample 3.”. However, do the authors checked if reducing the number of categories for an item could affect model fit? Secondly, was this procedure also applied in sample 2? If not, why? Thirdly, if this procedure was in fact applied in Sample 2, it was not in sample 1. Therefore, the first aim stated by the authors for this second study “The first aim was to determine whether the bifactor model that was found in Study 1 could adequately represent responses on the FTD-SS obtained in a second student sample, and to explore whether the fit of this model surpassed that of an alternative second-order model, as well as the correlated three-factor solution reported by Barrera et al., (60) and a simple unidimensional model.”, may not be demonstrated.***

***Lastly, since this procedure was not performed on the sample 1, the second aim indicated by the authors “The second aim was to explore the generalizability of the best fitting model by investigating the invariance of the confirmed factor solution in a third sample derived using a different recruitment strategy.” may not be demonstrated. The fit of a model with a reduced number of categories for an item cannot be compared to the fit of a model with no reduced item categories.***

***I suggest the authors to not reduce the number of categories and repeat the analyses.***

***- Line 324: The authors state “The best fitting of these models was then applied to the responses collected in Sample 3 to demonstrate form invariance across the two samples.”. However, it is not clear to which samples they are referring to? sample 1 and 2? or 2 and 3?***

***Moreover, they reported testing for “form invariance”. Do they perhaps mean configural invariance? I suggest the authors to explain that part better.***

***Anyway, I agree with the authors that is important to test the invariance of the model, however, I would suggest to compare males and females or to re-organize the analyses conducted on each sample (so maybe they could use two sample to test fo invariance), or to collect new data.***

We thank the reviewer for this feedback. Combining the samples in Study 2 has removed these problems and brought the analysis closer to its aim. The CFA has been re-conducted with all response categories for item 15 intact.

Unfortunately, we are not able to test configural invariance in this current data (Sample 2) because of insufficient data (i.e. unequal group sizes) across our demographics. We have noted this in the discussion as an important avenue for future research (page 42, lines 680-682).

***- Move “Study 3” (see comment for “Study 2”).***

We have moved the title, as suggested.

***- Moreover, since the aim of study 3 was to assess the validity of FTD-SS with other measure of psychopathology, it is not clear what is the rationale (or aim) behind the regression analyses conducted. I kindly ask the author to explain the need for regression analyses, otherwise I suggest the authors to drop these analyses. There are already multiple analyses in the manuscript, therefore adding analyses not supported by a clear aim could make the manuscript even harder to read.***

The underlying theoretical motivation behind the current study was that the FTD-SS might be a more sensitive measure of DCTS in schizotypy, when compared to other schizotypy measures that are typically used, because the FTD-SS might better capture the multidimensionality of DCTS that has been reported in clinical samples (pages 4-5, lines 74-86; page 7, lines 128-141). Thus, the aim of the study was to investigate the construct validity of the FTD-SS, with investigations of the dimensionality being a main focus (for Study 1 and Study 2). The regression analyses in Study 3 are, nevertheless, integral to the main theoretical motivation because they help evaluate how the FTD-SS performs in comparison to other measures of schizotypy. Notably, very few studies have investigated the convergent validity of self-report measures of DCTS, and our previous study is the only one to do so for the FTD-SS (Sumner et al., 2020). Since the schizotypy measures explained around half the variance in FTD-SS scores in the current dataset, and this convergence was not accounted for by neuroticism or extraversion (which have been suggested to contaminate self-report measures of DCTS), the findings of Study 3 should foster additional research into the potential incremental validity of the FTD-SS.

***-Line 409: It is not clear what do the authors mean with “A combination of free-response and multiple-choice questions were presented to record the following demographic information…”. I kindly ask the authors to explain better and rephrase.***

The wording has been removed to avoid confusion (page 29, lines 445-446).

**- Please add for each measure administered: (1) the scale (e.g., Likert scale from …); (2) what higher scores mean; (3) indices of internal consistency (e.g., Cronbach’s alpha or ordinal alpha).**

We had already calculated coefficient omegas to test internal consistency in Study 2, as recommended by several authors under most conditions (e.g. Reise et al., 2010; Zinbarg et al., 2005). However, for comparability, we have added total ordinal alpha coefficients for the FTD-SS in both Sample 1 (page 19, lines 260-261) and Sample 2 (page 27, lines 389-390). Ordinal coefficient alphas for the other scales were provided in the supporting information section, and these have now been moved into the manuscript (pages 30-32).

The response scales and interpretation of scale scores have also been added for all of the measures (page 8, lines 158-160; pages 30-32).

***- Since the authors reported that they reduced the number of categories for item 15 in for sample 2 and 3 in the second study, but not for sample 1 in the first study, and, moreover, since for study 3 they used pooled data from all the samples, it is not clear if they reduced the number of categories also for sample 1. However, I suggest the author not to reduce the number of categories (see previous comment) and repeat the analyses for study 3.***

We have combined the two samples in Study 2, removing the need to collapse the number of categories for item 15. Thus, all analyses are now consistent across all samples, including the analyses conducted in Study 3.

***General consideration: overall the manuscript is hard to read, mainly because its organization and the high number of statistical analyses. I suggest the author to re-organize the structure of the manuscript, by both re-organize the sections of the different studies, and computing the analyses on the whole sample. If the last suggestion is not possible, then I recommend the authors to re-organize the analyses conducted on each sample, without being too redundant.***

We again thank you for your constructive feedback and we hope that the clarifications made are sufficient to improve the readability of the analyses. Unfortunately, we cannot re-structure the analyses because, as mentioned, the CFA of Study 2 is based upon the findings from the EFA of Study 1, and because the analysis in Study 3 are based on the findings from Study 1 and Study 2 (i.e. continued support for the bifactor model). However, we have combined the sample for Study 2 and re-conducted the CFA, based on your feedback. Hopefully, with the edits made, you find the analyses easier to follow.

**Reviewer #2:**

***Thank you. This is a solid report, part of a consistent line of research of a scientific and clinical importance. The statistical and psychometric analyses are complex, sound, and well presented. The implications and potential developments of the findings are well discussed. Just a minor detail: there may be a typo in line 633.***

We thank you for your positive feedback, and for identifying the typo. This has been corrected.

**Additional References:**

(References used here but not in the manuscript)

Byrne (2010). Structural Equation Modelling with AMOS. Basic concepts, applications and programming. Second edition. New York, Routledge.

Zinbarg, R. E., Revelle, W., Yovel, I., & Li, W. (2005). Cronbach’s α, Revelle’s β, and McDonald’s ω*_H_*: Their relations with each other and two alternative conceptualizations of reliability. *Psychometrika*, *70*(1), 123-133.
