# Supplementary material for: Assessing the dimensionality of scores derived from the Revised Formal Thought Disorder Self-Report Scale in schizotypy
Source: PLoS One. 2022 Dec 9;17(12):e0278841. doi: 10.1371/journal.pone.0278841 (PMC9733900; doi:10.1371/journal.pone.0278841)
Supplement: Attachment — Submitted filename: Response to Reviewers.docx [file pone.0278841.s004.docx]

Prof. Marco Innamorati

Academic Editor

PLOS ONE

Resubmission of manuscript ‘*Assessing the dimensionality of scores derived from the Formal Thought Disorder Self-Report Scale in schizotypy*’

15^th^ July, 2022

Dear Professor Marco,

We would like to thank you for the opportunity to revise our manuscript, and to thank Reviewer 1 for their persistence in providing us with useful feedback. We hope that the research has been improved as a result, and that it will eventuate in a useful contribution to the literature.

Please find our responses to the reviewer’s comments below. For clarity, we have presented the comments in bold and in italics, and included a description of how and where (i.e. line numbers) the text was modified. As requested, changes made in the manuscript are marked using tracked changes. The amendments were discussed and agreed upon by all of the authors.

We eagerly await your reply regarding our revisions and are ready to respond to any further comments and questions that you may have.

Sincerely,

Philip Sumner, PhD

Swinburne University of Technology

Note. Line and page numbers are based on the manuscript displayed with tracked changes using “Simple Mark-up”.

***Reviewer #1: STUDY 1***

We thank Reviewer 1 for taking the time to go through our manuscript a second time, and offering their valuable feedback. We have tried our best to implement all suggestions in some way.

***- The authors state that they combined the second and third sample, which now is referred as “Sample 2”. I support this decision because there is no need to collect two more samples in order to assess replicability and generalizability of the model.
However, during the first review process I also suggested to the authors to assess whether there are socio-demographics differences between the samples. I did not find any tables or results showing whether the two samples are statistically different or not. So, again, I kindly ask the authors to conduct these analyses. If the two samples are statistically significant, I suggest the authors to repeat the EFA on both samples to assess whether these differences had affected the models whatsoever.
In any case, if there are statistical differences for socio-demographics, I suggest the authors to discuss the results in line of these differences.***

Between-group differences have now been tested and are presented in Table 1. We have also discussed the potential sample differences as potentially influencing the fit of the unidimensional model in the CFA with Sample 2, and so this is a study limitation (lines 538-546, page 30). Although we have not reported this, the EFA results in both samples were somewhat similar. Although the parallel analysis indicated 2 factors in Sample 2, Heywood cases were still evident in the multidimensional solutions. There were also similar signs of item redundancy in both samples, and the case for unidimensionality strengthened with the removal of these items in both samples (mentioned lines 360-375, pages 20 and 21).

Note too that we now discuss demographic influences and sampling characteristics extensively when comparing our findings with those of Barrera et al. (2015; see lines 425-478, pages 26 and 27).

***- I am not totally satisfied with the explanation given by the authors on the decision to keep the nine items in the test. If seven items did not load on any of the group factors, I suggest the authors to perform a deeper item analysis (e.g. Mokken analysis) and eventually remove these problematic items. The presence of seven problematic items is too big to ignore and decide that these items should still be included in the scale without a proper item analysis.
The same goes for the two items that did not load on the general factor.***

We agree that an item analysis is required but maintain that it is still important to show the performance of the original scale with all 29 items. As mentioned above, we discuss the differences in the item responses sampled in the current study (both Sample 1 and Sample 2) compared to those sampled by Barrera et al. (2015), despite seemingly similar recruitment avenues and sample demographics between the two studies. Reasons for these differences are not yet clear. However, it makes sense to present this evidence and the inability to replicate their correlated three factors model before revising the scale, since the measure has already been created and used.

Nevertheless, there were some signs of item redundancy in both samples. We have now reported the largest inter-item polychoric correlations that we found in Sample 1, and note that these items also produced large residual correlations across all dimension-reduction techniques in both samples. We then indicated the effect of removing these items on the evidence in support of unidimensionality (see lines 354-375, pages 20 and 21). This promotes the conclusion that revisions to the scale are necessary, at least in samples with levels of endorsement comparable to those in the current study.

Moreover, based on your feedback below, we have revised the description of the results from the bifactor model (and the added Rasch analysis) to indicate that we are evaluating the evidence in support of the plausibility of subscales (i.e. multidimensionality) and the evidence in support of the plausibility of the overall score (i.e. unidimensionality; following the approach outlined by Dunn et al., 2020). In this context, the high number of items that did not load significantly on any group factor was taken as support for unidimensionality. On the other hand, the two items with non-significant loadings on the general factor, and the poor fit of the unidimensional model in Sample 2, both suggest the need for item revisions.

We have elaborated our point in the discussion that some of the FTD-SS items do not seem to be appropriate for non-clinical samples, at least in our non-clinical samples, given that some items exhibited low levels of endorsement (high positive skew). We then suggest that a deeper item analysis be conducted in future studies. We hope to conduct these analyses as a separate manuscript, using the Rasch analysis more extensively (beyond testing the assumption of unidimensionality).

***- The authors state “Because the factors found in the initial analysis appeared to be highly correlated with one another (≥ 0.56), a follow-up exploratory linear bifactor model was conducted…”.
However, this is not in line with what a bifactor model aims at assessing. A bifactor model aims at assessing unidimensionality: when the items report a greater load on the general common factor than on the other group factors, than it can be assumed that the construct is essentially unidimensional, and this is not the case for the bifactor model reported in the manuscript. Some items show a greater loading on the general common factor, while others show a greater factor loading on the other factors extracted.
Moreover, Reise (2012) reported “Exploratory analyses allow researchers to identify potential modeling problems directly rather than indirectly through post hoc inspection of fit and modification indices after estimating a confirmatory model.” (Reise, S. P. (2012). Invited paper: the rediscovery of bifactor measurement models. Multivariate Behav. Res. 47, 667–696. doi: 10.1080/00273171.2012.715555). Which means that a bifactor model cannot be the final solution. For these reasons, I suggest the authors to use the bifactor model to identify the presence of problematic items (see previous comment), and to modify the model if needed (since two items did not basically load on the general common factor (0.15 and 0.24)).
Moreover, the author state “The addition of a fourth factor in the bifactor model explained an extra 4.72% of the variance ”. That is obviously true because the more factors there are, the more variance is explained, and that is why we have to be careful with factor analysis in trying to find a balance between the number of factors and the variance explained. Including a general common factor, in addition to the three group factors already extracted, is not a valid theoretical solution. A general common factor cannot be considered, for example, as a second-order factor, which can be used for theoretical explanation.
Lastly, the authors state that in the CFA all items significantly loaded on the bifactor model. However, if the EFA did not indicate a good fit to the data, then it is pointless to perform a CFA (since the aim of the CFA is to confirm the results found by the EFA). So, I kindly suggest the authors to reconduct the EFA after the analyses on the problematic items.***

We thank the reviewer for this feedback. The description of our findings has been revised to better indicate evidence for and against unidimensionality, and removed mistaken statements suggesting that the bifactor model was a final solution. We had already concluded that the best evidence from the bifactor model was of essential unidimensionality (a conclusion that was confirmed by the corresponding author for the FACTOR program when I wrote to him to clarify the output), suggesting that a total/overall score would be the most meaningful measure derived from the questionnaire. We had initially based this on the closeness-to-unidimensionality indices that are presented as supporting information. We have now bolstered this conclusion by further considering the pattern of factor loadings in the bifactor model, conducting a Rasch analysis, and assessing the influence of item redundancy. We have also scaled back the CFA to simply show that the unidimensional model was a poor fit in a second sample. Perhaps this is unsurprising, though it serves to reproduce the lower levels of item endorsement that contrast the data presented by Barrera et al. (2015). It also affirms the need for a more in-depth analysis of the items and revisions of the scale.

***- Because of the previous comment, I kindly suggest the authors to remove the bifactor model fit from Table 3. Table 3 should be a recap of all the possible model solutions (since it was established that a bifactor model cannot be considered a model solution). For model comparison the authors could report the AIC and BIC values and choose the most suitable model on the basis of their values.***

We have removed Table 3. As you mentioned above, it makes little sense to test models that were not supported by the EFA. Since the balance of evidence from the exploratory analyses favoured a unidimensional model, this is the only model now tested using the CFA. Although the figures depicting the unidimensional model, Barrera et al.’s correlated three-factor model and the bifactor model have been retained, these are for illustrative purposes.
 ***STUDY 2
- “Line 322: I do not fully understand the rationale behind testing a second-order model on the bifactor model. Could the authors explain this decision?” Response: “Indeed, the two models appear similar, both representing three group factors and a general factor. However, the two models differ in the specified relationships between the group and general factors, and so yield different conceptual representations of multidimensionality (see Reise et al., 2010). In the bifactor model, direct relationships are modelled between the observed item variance and the general factor, and the group factors explain additional item variance that is not accounted for by the general factor. One intuitive interpretation of the group factors is that they represent nuisance variables that interfere with the measurement of the general factor (Reise et al., 2010). By contrast, in the second-order hierarchical model, the general factor is not directly related to the item variances. Instead, the general factor accounts for the variance that is common to the group factors.
We attempted to explain this distinction in the discussion for Study 1 using intelligence models as an example. The explanation has been re-worded slightly in an effort to clarify the rationale (page 21, lines 301-305, 310-312). The differences between the models are also depicted in Figures 3 and 4. ”

I know what a second-order hierarchical model is, however, that is not what I asked when I made the comment on the first review process. In the original version of the manuscript it was reported that new models were tested on sample 2, among which “… and a second-order model based on this bifactor model”(former line 322). Maybe it was the wording that was misunderstanding or the fact that the manuscript was (and still is) hard to read, but it seemed like that a second-order bifactor model was being tested, and that is pointless to test.***

These sections have been removed.  ***- The authors replied to my comment saying that they were unable to test configural invariance because of insufficient data, so I wanted to know what kind of invariance they tested? What is “form invariance”? Please, could the authors provide some information?***

“Form invariance” was used erroneously. We had hoped to explore whether the factor solutions differed across potentially important demographic variables (e.g. gender). Originally, the second sample was collected using REP and the third sample was collected using Prolific (these groups are now combined into Sample 2). Prolific was used for recruitment in the hope of collecting a more representative non-clinical sample, including a greater proportion of males and less students. The first step is to test configural invariance, before successively constraining model parameters to be equal to test stricter forms of invariance. However, our sample sizes were not large enough to test invariance between levels of any the demographic variables assessed.

***General consideration: I kindly ask the authors to re-organize the manuscript in light of the comments made about the bifactor model. Moreover, I kindly suggest the authors to take into consideration the fact of rearranging the manuscript order on the basis of the results of the analyses requested in the first 2 comments (the analyses on the differences in socio-demographic variables between the two samples was already requested during the first review process). Hence, one possible solution could be to combine Study 1 and Study 2 (since the main analysis conducted is factor analysis), which will become “Study 1” and then Study 3, which will become “Study 2”. If the authors do not agree with this suggestion, I kindly ask them to add the new analyses in the most suitable place in the manuscript so it will not make it harder to read.***

We have now re-structured the manuscript, removing the Study 1/Study 2/Study 3 format and organising the results by “exploratory dimension-reduction analyses”, “confirmatory factor analysis”, “potential item redundancy”, and “convergent relationships with schizotypy and the influence of demographics”. The between-group comparisons have been provided in Table 1.

***Reviewer #2: (No Response)***
