# Supplementary material for: Assessing the dimensionality of scores derived from the Revised Formal Thought Disorder Self-Report Scale in schizotypy
Source: PLoS One. 2022 Dec 9;17(12):e0278841. doi: 10.1371/journal.pone.0278841 (PMC9733900; doi:10.1371/journal.pone.0278841)
Supplement: Attachment — Submitted filename: Response to Reviewers.docx [file pone.0278841.s005.docx]

Prof. Marco Innamorati

Academic Editor

PLOS ONE

Resubmission of manuscript ‘*Assessing the dimensionality of scores derived from the Formal Thought Disorder Self-Report Scale in Schizotypy*’

31^st^ October, 2022

Dear Professor Marco,

We would like to thank you for the opportunity to revise our manuscript, and to thank the reviewer for their continued patience and constructive feedback. Their advice has helped us navigate a difficult analysis, producing findings that we hope will eventuate in a useful contribution to the literature.

Please find our responses to the reviewer’s comment below. For clarity, we have presented the comments in bold and in italics, and included a description of how and where (i.e. line numbers) the text was modified. As requested, changes made in the manuscript are marked using tracked changes. The amendments were discussed and agreed upon by all four authors.

We eagerly await your reply regarding our revisions and are ready to respond to any further comments and questions that you may have.

Sincerely,

Philip Sumner, PhD

Swinburne University of Technology

Note. Line and page numbers are based on the manuscript displayed with tracked changes using “Simple Mark-up”.

**Reviewer #1*:***

***I appreciate the authors responsiveness to my comments. However, I still a have a minor concern which was not addressed in this second review process.***

***The authors provided support for a unidimensional factor structure for the FTD-SS with a bifactor model, however two items did not load on the general common latent factor. Moreover, the authors still decided to keep the seven items which did not load on the specific group factors. I was wondering if the authors tried to conduct the analyses without those item. Firstly, without all the nine items, then excluding the two items which did not load on the general common factor, and lastly excluding only the seven items which did not load on the specific group factors. By doing so it's possible to assess the changes in the factor structure, and, perhaps, an improvement of the model fit.***

***I think this is an important step in order to justify the presence of these items in the scale.***

We thank the reviewer yet again for their advice and patience. They were correct. We removed the items and repeated the factor analyses as suggested. The removal of items improved the sampling adequacy of the polychoric correlation matrix, and parallel analyses continued to indicate the presence of three factors. Nevertheless, it was only when all nine items were removed that the three-factor solution essentially achieved simple structure (see the revised Table 2 and Table C, S1 Supporting Information).

As mentioned in the previous re-submission, we were hesitant to remove items from the scale. The FTD-SS items were originally created by Barrera et al. (2008) based on previous descriptions of symptoms of formal thought disorder in the literature. A preliminary pool of 52 items were then reduced to the final 29 items based on an ‘item analysis’ of responses obtained from a sample of people diagnosed with chronic schizophrenia, although the authors admittedly did not describe this item analysis in much detail. All 29 items had obviously also survived in Barrera et al.’s (2015) principal components analysis of responses in their non-clinical.

With respect to this hesitancy, we brought back Barrera et al.’s (2015) model with all 29 items for the confirmatory factor analysis in Sample 2. We were then able to demonstrate the fit of the three-factor model with 20 items compared to Barrera et al.’s model with 29 items, as well as unidimensional models with both 20 and 29 items (see revised Table 3, p. 20). We are, therefore, satisfied that the removal of these items was justified.

We also re-visited our removal of items due to potential item redundancy. The inter-item correlations alone in Sample 1 were not necessarily high enough to justify removing the items straight away. However, in the last re-submission, we had removed three items (1, 9 and 14) with high inter-item correlations because they also showed high residual correlations in the Rasch analysis. As might be expected, this strengthened evidence in support of unidimensionality (although the fit of the unidimensional model was still poor with these items removed in the second sample). In an effort to strengthen the evidence of item-redundancy and better justify the removal of these items, we used Ferrando et al.’s (2022) method of detecting doublets based on residuals in the exploratory factor analysis, which is now implemented in an updated version of FACTOR. Only one pair of items (items 9 and 10) were identified as potential doublets. However, the removal of one of these items alone was not sufficient to support unidimensionality. Because the justification for the removal of the three items was not a strong as the removal of the nine items based on the bifactor model, we have moved it to the supporting information section and only mentioned it only briefly in-text (see Table C, S1 Supporting Information).

All of the exploratory factor analyses conducted, including those repeated after the removal of items, has now been presented systematically in a table within the supporting information section (see Table C, S1 Supporting Information). This includes the improvement in sampling adequacy, any changes to the results of the parallel analysis for determining the number of factors to extract, the fit indices of the resultant solution, and the presence of any cross-loadings. The loadings for the 20-item three factor model in Sample 2 derived using confirmatory factor analyses have also replaced those of the unidimensional model in the supporting information section (Table G).

Notably, the 20-item three-factor solution produced factors that were strongly correlated with one another, and closeness-to-unidimensionality indices still supported the use of total scores (see S1 Supporting Information). Moreover, the convergent correlation analyses were almost identical (*r*±0.02) when FTD-SS total scores were calculated from the 20 items instead of all 29 items. Thus, minimal changes were needed to the remainder of the analyses. Convergent bivariate correlation analyses were repeated with scores from the 20-item three-factor model, and these are presented in the supporting information (Table H).

In-text, there have been several minor changes throughout the manuscript to reflect the amended factor analyses. The loadings for the exploratory bifactor model have been moved from Table 2 to the supporting information (Table A) and replaced in-text by the loadings for the final 20-item exploratory factor solution (p. 19). Figures 1, 2 and 3 have also been altered to reflect the new confirmatory factor analyses, and a fourth figure has been added (see captions p. 31). Minor changes to the abstract (p. 2; lines: 16-23), and the order and content of the discussion (p. 25-30; lines: 419-450, 475-478, 496-498, 548-552) have been made to reflect the tipping of evidence in favour of multidimensionality. The conclusions remain largely the same, however, that the summed total score remains an important score derived from the FTD-SS, and that additional item-analyses are needed in the future.

Again, we would like to thank the reviewer for their suggestion.
