# Supplementary material for: Assessing the dimensionality of scores derived from the Revised Formal Thought Disorder Self-Report Scale in schizotypy
Source: PLoS One. 2022 Dec 9;17(12):e0278841. doi: 10.1371/journal.pone.0278841 (PMC9733900; doi:10.1371/journal.pone.0278841)
Supplement: Attachment — Submitted filename: Response to Reviewers.docx [file pone.0278841.s006.docx]

Prof. Marco Innamorati

Academic Editor

PLOS ONE

Resubmission of manuscript ‘*Assessing the dimensionality of scores derived from the Formal Thought Disorder Self-Report Scale in schizotypy*’

11^th^ November, 2022

Dear Professor Marco,

We thank both you and the reviewer again. The final suggestion was to amend the title of the manuscript to indicate that it was a revised version of Barrera et al.’s (2015) Formal Thought Disorder - Self-Report Scale (FTD-SS) that was investigated in the current study. The revision refers to the exclusion of nine items from the original 29-item measure, which was necessary before an adequate factor solution could be found.

Once we had finally acquiesced to the reviewer’s suggestion to remove items and repeat the exploratory factor analysis, we ourselves considered whether we should subsequently refer to the scale as a revised FTD-SS. However, we decided not to for two reasons.

Firstly, as we have stated in the discussion of the manuscript, the demographic characteristics of the two samples in the current study were comparable to those reported by Barrera et al. (2015). Yet, the average FTD-SS total scores obtained in our samples appeared to be somewhat smaller than those of Barrera et al.’s sample, from which their three correlated factors solution was found. The reasons for this are currently unclear. Therefore, we believe that future research should conduct Rasch analyses to further investigate the performance of the items of the FTD-SS in non-clinical samples, and that a revised scale should be produced in-light of this analysis and the results of the current study.

Secondly, subsequent analyses exploring the convergent validity of FTD-SS scores and the influence of demographic variables upon these scores were performed in the current study using total scores calculated from all 29 items. Repeating these analyses with scores derived from the revised 20-item scale made very little impact on the results. However, we left these analyses as they were for the reason mentioned above. Thus, it is more accurate to leave the title as it is.

We hope you find this acceptable and look forward to hearing from you shortly.

Sincerely,

Philip Sumner, PhD

Swinburne University of Technology

***Reviewer #1: I thank the authors for being responsive to my comments.***

***Just a minor suggestion regarding the title. Since some items have now been removed, perhaps writing "Assessing the dimensionality of scores derived from the Formal Thought Disorder Self- Report Scale - Revised (FTD-SS-R) in schizotypy" could be more appropriate. But I will leave the decision to the authors.***

Once we had finally acquiesced to the suggestion to remove items and repeat the exploratory factor analysis, we ourselves considered whether we should subsequently refer to the scale as a revised FTD-SS. However, we decided not to for two reasons.

Firstly, as we have stated in the discussion of the manuscript, the demographic characteristics of the two samples in the current study were comparable to those reported by Barrera et al. (2015). Yet, the average FTD-SS total scores obtained in our samples appeared to be somewhat smaller than those of Barrera et al.’s sample, from which their three correlated factors solution was found. The reasons for this are currently unclear. Therefore, we believe that future research should conduct Rasch analyses to further investigate the performance of the items of the FTD-SS in non-clinical samples, and that a revised scale should be produced in-light of this analysis and the results of the current study.

Secondly, subsequent analyses exploring the convergent validity of FTD-SS scores and the influence of demographic variables upon these scores were performed in the current study using total scores calculated from all 29 items. Repeating these analyses with scores derived from the revised 20-item scale made very little impact on the results. However, we left these analyses as they were for the reason mentioned above. Thus, it is more accurate to leave the title as it is.
