# Supplementary material for: Assessing the dimensionality of scores derived from the Revised Formal Thought Disorder Self-Report Scale in schizotypy
Source: PLoS One. 2022 Dec 9;17(12):e0278841. doi: 10.1371/journal.pone.0278841 (PMC9733900; doi:10.1371/journal.pone.0278841)
Supplement: Attachment — Submitted filename: Response to Reviewers.docx [file pone.0278841.s007.docx]

Prof. Marco Innamorati

Academic Editor

PLOS ONE

Resubmission of manuscript ‘*Assessing the dimensionality of scores derived from the Revised Formal Thought Disorder Self-Report Scale in schizotypy*’

22^nd^ November, 2022

Dear Professor Marco,

We thank both you and the reviewer yet again for your patience and continued feedback. Although this round of suggestions has had minimal impact on the results and conclusions of the study, the feedback has helped improve the consistency of the approach taken throughout the manuscript.

As usual, please find our responses to the reviewer’s comments below. For clarity, we have presented the comments in bold and in italics, and included a description of how and where (i.e. page and line numbers) the text was modified. As requested, changes made in the manuscript are marked using tracked changes. The amendments were discussed and agreed upon by all four authors.

We hope you find this acceptable and look forward to hearing from you shortly.

Sincerely,

Philip Sumner, PhD

Swinburne University of Technology

Note. All page and line numbers are based on tracked changes using “Simple Markup”.

***Reviewer #1: I am sorry but I cannot address the publication of the manuscript yet.***

***In the previous revisions I suggested the authors to perform changes on the factor model of the FTD-SS and to consequently adapt the results of the other analysis. The authors deleted 9 nine items from the questionnaire, which improved the fit of the model, however still kept those nine item to compute total score. How can this be correct? The readers could ask why those items were used if they were not included in the model? What is the portion of the variance explained by those items? Why did the authors not want to remove those items from the total score? The decision to still keep the nine items cannot just be theoretical. If the analyses suggest that these items should be removed, then it is pointless to compute total score with those items because they do not contribute to the factor model at all. Total score needs to be computed from only the items included in the model.***

***If the authors wish to compute two different total scores (one with 20 items and the other with 29 items) and analyze whether there are any statistical differences, then it is ok, but they cannot use all 29 items when the model suggested the inclusion of only 20 items.***

We agree that it is better to have a more consistent approach throughout the manuscript. In fact, we did previously analyse total scores calculated both from the revised 20-item scale and the original 29-item scale, and the results of the bivariate correlation analyses between scores from the 20-item version of the scale and the other schizotypy measures were presented in S1 Supporting Information. Our failure to change the multiple regression analyses and ANOVAs in the main manuscript was merely a matter of convenience, since repeating the analyses with total scores computed from the revised 20-item scale had negligible impacts on the results.

In particular, the effect sizes of all zero-order correlations, partial correlations, standardized regression coefficients, and *R*^2^ values for the 20-item FTD-SS-R total scores in both of the regression models (to investigate convergence with the SPQ and O-LIFE schizotypy scales) were all within 0.03 of those for the 29-item FTD-SS total scores. Indeed, we found that the correlation between total scores calculated from the 20-items and the 29-items were very large, and the changes to the analyses with demographic variables were also largely inconsequential.

As you had suggested previously, we now refer to the 20-item scale as the revised FTD-SS (FTD-SS-R). This change is evident in the title (p. 1) and abstract (lines 20-21, p. 2), and in the results (lines 355-356, p. 19; Table 3, p. 21; lines 384-428, p. 23-24; Table 4, p. 25; Table 5, p. 26) and discussion (e.g. lines 436 – 445, p. 27). Note that, where we refer to both the FTD-SS and the FTD-SS-R, such as when making comparisons in the discussion, we include the number of items (e.g. “20-item FTD-SS-R”) for additional clarity.

We have also repeated all of the analyses conducted after the EFA and CFA so that they are performed with total scores from the 20-item FTD-SS-R. Specifically, all effect sizes, *p* values and confidence intervals have been re-calculated for both the multiple regression analyses (see Table 4, p. 24) and ANOVAs (see Table 5, p. 26). However, because we discuss the demographic similarities between the samples of the current study and Barrera et al.’s (2015) sample, and the influence of demographics on the FTD-SS scores in these studies, we have moved the original table of ANOVAs based on the 29-item scale to S1 Supporting Information (Table I) rather than remove it entirely. Finally, the very minor differences between the results of analyses conducted on total scores from the original 29-item scale have also been described (lines 403-414, p. 23; lines 427-428, p. 24).
